# Kazimierz Orzechowski (1878**–**1942)

**DOI:** 10.1007/s00415-017-8724-4

**Published:** 2017-12-30

**Authors:** Anita Maria Magowska, Michał K. Owecki, Halina Bogusz

**Affiliations:** Poznan University of Medical Sciences, Poznan, Poland

Kazimierz Orzechowski, a Polish neurologist, coined the term ‘opsoclonus’ to refer to unpredictable involuntary, horizontal and vertical oscillations of the eyes occurring in some neurological disorders [1]. While today the term is associated primarily with the opsoclonus–myoclonus syndrome, first described in 1962, it is still frequently used in its original meaning, as confirmed by 38,618 articles in the PubMed database (on 30 October 2017).

Kazimierz Orzechowski was born into a noble family in the town of Przemyśl in the Austrian Partition (since the end of the eighteenth century to 11 November 1918 Poland was partitioned by Austria, Prussia and Russia) on 5 February 1878. After graduating from secondary school, he went to Lvov (also in the Austrian Partition), where he studied medicine. Having obtained a doctor’s diploma in 1902, he worked for several months as an intern in a local hospital, but soon went to Vienna to learn about the emerging subject of neurology. He was admitted as an intern to the Second Medical Clinic of the University of Vienna which was run by Edmund von Neusser (1852**–**1912). At the same time, he worked at the Clinic for Psychiatry and Nervous Diseases which was led by Richard von Krafft-Ebing (1840**–**1902) and, after his sudden death, by Julius Wagner-Jauregg (1857**–**1940), a pioneer in the study of the effects of thyroid diseases on the nervous system.

A year later, in 1904, he moved to the Neurological Institute which was founded and run by Heinrich Obersteiner (1847**–**1922), a prominent neuroanatomist and neuropathologist, at the University of Vienna. There he carried out his first histological examination of neoplasms in the subarachnoid space and presented results at the meetings of the Psychiatric Society in Vienna. In 1907, he also worked at a clinic led by Lothar von Frankl-Hochwart (1862**–**1914), who conducted innovative research on pituitary disorders, the pathogenesis of tetany and brain tumours.

In February 1909, rich in knowledge and skills, he returned to Lvov to head a much-neglected department of mental diseases at the city hospital. In a short time, he transformed it into a well-organised neurological department with an adjacent clinic. In 1910, he received a postdoctoral degree (called in German-speaking countries ‘habilitation’) in the anatomy of the nervous system and was appointed as an assistant professor of neuropathology without the right to remuneration. He also conducted all histological examinations at the department, trained other doctors in this field and took an active part in the meetings of the Lvov Medical Society.

**Fig. 1:**
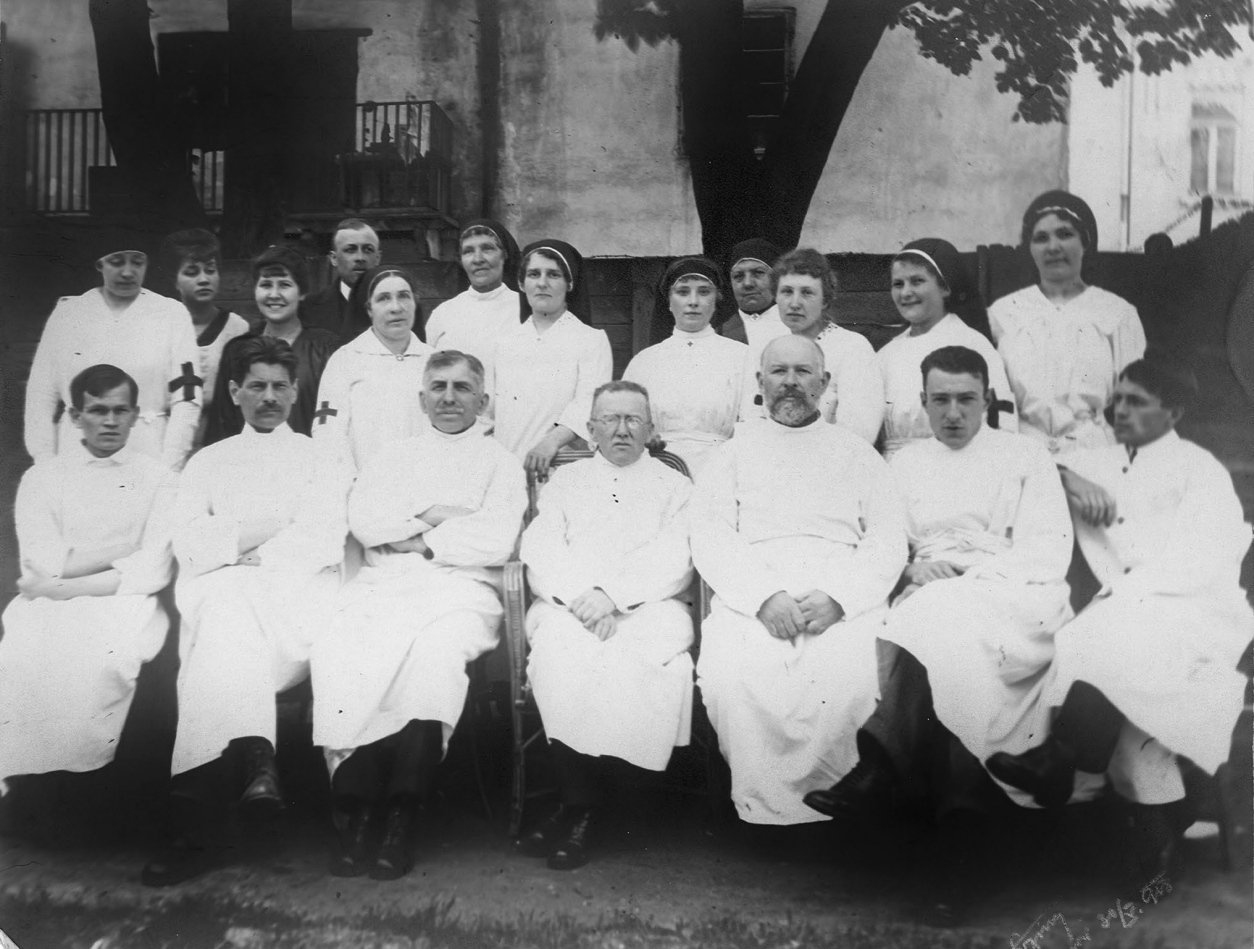
Medical staff of the Red Cross Hospital in Lvov in 1917, Kazimierz Orzechowski is in the middle of the lower row (photo from the author’s collection)

After the outbreak of World War I, he became a neuroscience consultant at the Red Cross Hospital in Lvov and headed the department of nervous system diseases for some time. After the end of this war, he was appointed as an associate professor of neurology at the University of Lvov (Fig. 1).

At the beginning of the twentieth century, Lvov was the most important medical centre on the territory of Poland (still partitioned) since all local doctors were graduates or former employees of the University of Vienna. No wonder then that, after Poland regained its independence in November 1918, newly established or reactivated Polish universities sought candidates for chairs and clinics there. In the spring of 1920, being a full professor, Orzechowski was appointed as organiser and head of the Department and Clinic of Neuroscience at the University of Warsaw. The newcomer from Lvov faced open hostility from Warsaw neurologists, the followers of Edward Flatau (1868–1932), who were convinced that the prestigious clinic should be entrusted to one of them. After the death of Flatau in 1932, Orzechowski took over his legacy and became the head of the Department of Neurobiology at the Nencki Institute of Experimental Biology and the Neurosurgical Department at the Trauma Institute in Warsaw.

For the rest of life, he remained as the most influential figure in Polish neurology. He founded the Warsaw Neurological Society, later transformed into the Polish Neurological Society, and became its president. He also established its organ *Neurologia Polska* [*Polish Neurology*] and from 1938 was its editor-in-chief. In 1928, he founded the Polish Society for Brain Research and became its president too [2]. He soon led to the founding of the Brain Research Institute in Warsaw and entrusted it to Maksymilian Rose (1883–1937) [3]. When Marshal Józef Piłsudski (1867–1935), the most important politician in interwar Poland, died, Orzechowski performed an anatomopathological examination of his brain.

The righteous character, tactful behaviour and his constant willingness to help others attracted younger colleagues to Orzechowski to obtain doctoral degrees under his supervision. Among them were excellent Polish neurologists, such as Jerzy Choróbski, Łucja Frey, and Adam Opalski. For some time, he was the Dean of the Faculty of Medicine in Warsaw [2].

Orzechowski was one of the most prominent European neurologists, confirmed by his membership in foreign scientific societies and nearly a hundred publications containing innovative scientific ideas, which were later borrowed and developed by others who often forgot about the author. For example, in 1913, he described a case of a 21-year pregnant woman with the cyst of the cerebellum and the opsoclonus syndrome. He never thought to measure the eye movements and was satisfied with perimetric evaluation of visual field defects. After two surgeries, the woman died unexpectedly and the autopsy showed inflammation of the cranial pia mater as the cause of the cyst [1]. In 1927, Orzechowski reported the association of opsoclonus with myoclonus; however, Kinsbourne’s description of opsoclonus–myoclonus from 1962 became classic [4]. He had published an article about the diagnosis of hysteria with the symptoms of tetany before Hans Curschmann (1875–1950) published similar diagnostic criteria [5]. In his work written together with Witold Nowicki, Orzechowski had demonstrated, for the first time, the anatomical similarity of von Recklinghausen’s disease (neurofibromatosis type I) and tuberous sclerosis, which was confirmed by the ophthalmologist Jan van der Hoeve (1878–1952) several years later.

Orzechowski questioned the thesis that spinal nervous centres disappear after the muscular connection with the nervous system is broken, i.e. after amputation. His idea was then taken up by Hermann Oppenheim (1858**–**1919) in the third edition of his textbook entitled *Lehrbuch der Nervenkrankheiten für Ärzte und Studierende* [*A Textbook of Nervous Diseases for Physicians and Students*]. Orzechowski also overthrew the thesis popular in old neurology that the central nervous system is unable to regenerate. He thus became the forerunner of the concept of the brain’s neuroplasticity, which was taken up by Alois Alzheimer (1864**–**1915) and later by others, including Max Bielschowsky (1869**–**1940) and Otto Marburg (1874**–**1948) [6].

Before genetics was established, Orzechowski proved that neurological diseases could be hereditary. He was the first to show that otosclerosis (also known as otospongiosis) is inherited [7]. He noticed that innate predispositions for endocrine disorders affect the progression of epilepsy and syphilis of the nervous system. He was also the first to use diets to affect endocrine activity in order to ease the progression of epilepsy [8].

His works also included a wide range of publications devoted to neoplastic diseases. He co-authored with Zygmunt Kuligowski (1902**–**1998) the first description of a rare form of the brain tumour—neuroblastoma verum of the frontal lobe. Marburg and Oppenheim also cited his work about the inherited tendency to spinal neuroblastomas [9]. He paid much attention to the pathology of the muscular system. In 1909, he described a paroxysmal symptom myotonia, i.e. a short-lasting muscle weakness after a myotonic contraction. He then analysed all the available literature demonstrating cases of atrophic and nonatrophic myotonias to demonstrate that the former was indeed an inherited disorder [10].
